# Microsaccadic rate signatures correlate under monocular and binocular stimulation conditions

**DOI:** 10.16910/jemr.13.5.3

**Published:** 2020-08-11

**Authors:** Peter Essig, Alexander Leube, Katharina Rifai, Siegfried Wahl

**Affiliations:** Institute for Ophthalmic Research, Eberhard Karls University Tuebingen, Germany; Carl Zeiss Vision International GmbH, Aalen, Germany

**Keywords:** Eye movements, Microsaccades, Fixation, Visual performance

## Abstract

Microsaccades are involuntary eye movements occurring naturally during fixation. In this study, microsaccades were investigated under monocularly and binocularly stimulated conditions with respect to their directional distribution and rate signature, that refers to a curve reporting the frequency modulation of microsaccades over time. For monocular stimulation the left eye was covered by an infrared filter. In both stimulation conditions, participants fixated a Gabor patch presented randomly in orientation of 45° or 135° over a wide range of spatial frequencies appearing in the center of a monitor. Considering the microsaccadic directions, this study showed microsaccades to be preferably horizontally oriented in their mean direction, regardless of the spatial characteristics of the grating. Furthermore, this outcome was found to be consistent between both stimulation conditions. Moreover, this study found that the microsaccadic rate signature curve correlates between both stimulation conditions, while the curve given for binocular stimulation was already proposed as a tool for estimation of visual performance in the past.

Therefore, this study extends the applicability of microsaccades to clinical use, since parameters as contrast sensitivity, has been measured monocularly in the clinical attitude.

## Introduction

Fixation on a visual target is not stable, instead such a fixation is accompanied by small involuntary eye movements – fixational eye movements (FEMs) (1, 2, 3, 4, 5, 6). Among FEMs, three types of eye movements are included as tremor, drift and microsaccades (7, 8), which are distinguished from each other by their amplitude and velocity (for review see 8, 9, 10, 5).

Microsaccades are very fast FEMs (approximate range of velocities 50–200 deg/s) with a typical amplitude smaller than 1° (11) and a rate of 1–3 Hz (8, 12). In the past years, microsaccades have been discussed for their potential impact on vision. Researchers have shown that microsaccades optimize gaze position in high visual acuity tasks (13), as well as they enhance visual acuity by optimizing the image position on the retina (14). Microsaccades have been also found to be linked with covert attention (15, 16, 17). Moreover, past research showed microsaccades as an indicator for discrimination of the orientation of a contrast stimulus featured by higher spatial frequency, however not for the stimulus of lower spatial frequency (18). In addition, those fixational saccades indicated sensitivity in the rate signature curve for small changes in contrast using a spatially oriented pattern with fixed spatial frequency of 0.33cpd (cycles/degree) (19), as well as for larger changes in contrast using a spatially oriented grating with fixed spatial frequency of 3.0cpd (20). Furthermore, it has been shown that the microsaccadic rate signature is sensitive to changes in luminance and contrast in colour of a circular visual stimulus, or to presence of an auditory stimuli (2). In terms of microsaccadic orientation, it has been disclosed that microsaccades occur in the spatial direction in which the attentional cue appears (16, 21). Additionally to this finding, microsaccades were found to be predominantly leftwards oriented in reading tasks and thus helping to refine vision by correction of inaccuracies in saccadic landing and by moving the gaze over the nearby words (22). The microsaccadic directional distributions have also been demonstrated to vary for binocular and monocular microsaccades, using the EyeLink II eye tracker (23). However, the search for purely monocular microsaccadic events using Dual Purkinje Image eye-tracker and magnetic induction eye-coils failed (24). Regarding to this discrepancy, as it has been shown, the term of monocular microsaccades was understood differently across the studies (for review see 20, 25, 26). Accordingly, as proposed by Nyström, Andersson, Niehorster, & Hooge, (27); Otero-Millan et al., (1); Otero-Millan, Troncoso, Macknik, Serrano-Pedraza, & Martinez-Conde, (28); Engbert & Kliegl,(29) , the current study followed the understanding of microsaccades as a strictly binocular phenomenon. Despite these findings, microsaccades have been shown to occur in both, monocularly and binocularly stimulated conditions by stimulating either under binocular viewing conditions, or randomly left and right eye, while the participant perceived blank space with the fellow eye (30).

Previously performed experiments on monocularly stimulated microsaccades have shown some methodological limitations, as for instance performing the separation of the visual input in monocular stimulation just by presenting different visual stimuli to both eyes. Therefore, this approach may result in a not totally-separated visual stimulation and thus lead to imperfect monocular stimulation and to methodological inconsistencies between outcomes of the different literatures. In this relation, the current study protocol proposed a distinct monocular visual stimulation condition by coverage of one eye with an infrared filter. Consequently, without visual stimulation of that eye but allowing the eye tracker to capture the eye movements binocularly.

According to the previous observations, the current study targeted the question, whether both, monocular and binocular stimulation of microsaccades by a spatially oriented pattern will result in a comparable rate signature curves. The expectation of the current study was that the rate signature curves will correlate under the two distinct stimulation conditions, as long as the Hering’s law of equal innervation (31) was taken into account in both circumstances. In addition, recent findings of Hafed, Goffart, & Krauzlis, (32); Hafed & Krauzlis, (33) indicated the same neural circuit for production of microsaccades and normal saccades. Although, it seems that microsaccades coming from both monocular and binocular fixation share the same neural origin as they are understood as a conjugate eye movements (34, 35), there is an unsatisfying research questioning, whether the visual performance correlation with microsaccades can provide clinically meaningful measures. On the one hand Bonneh et al., (20) and Scholes et al., (19) proposed the microsaccadic rate signature as a reliable estimator of contrast sensitivity, however, measured only under binocularly stimulated conditions. On the other hand, Denniss, et al. (36) measured contrast sensitivity under monocularly stimulated conditions, however the actual comparison of the microsaccadic rate signatures under distinct stimulation conditions was not considered. As the standard clinical measurements of contrast sensitivity are performed under monocular viewing conditions (37), the main aim of the current research was to establish a methodological approach of a monocularly stimulated microsaccadic rate signature in healthy subjects with appropriate comparison to classical binocular stimulation of microsaccades. This attitude could be used as a visual performance indicator in the future, following the clinical standard as already proposed by Denniss et al.,(36).

For the theoretical motivation, pushing the understanding of microsaccadic occurrence forward, this study also questioned whether the monocular and binocular stimulation will result in the same descriptive features of a microsaccadic rate signature. Thus, by showing correlated rate signature curves in both stimulation conditions, this study proposes that microsaccades, triggered by either monocular or binocular external visual input, should be rather taken as the same physiological phenomenon. Therefore, the current research provides an additional information for better future understanding the generation of those fixational eye movements.

Next, the current study investigated whether the condition of monocular stimulation will have any influence on the mean direction of microsaccades. In addition, it was examined whether a low level spatial characteristics of a centrally located visual stimuli will change the distribution of microsaccadic directions and thus will provide an information about the spatial characteristics of such a visual stimulus. This parameter could be then potentially used as an additional indicator of visual sensitivity.

Accordingly, the first hypothesis assumed, that the majority of microsaccades will follow the orthogonal direction of a presented Gabor patch, since micosaccades have been shown to be potentially visual input dependent (18, 19). The highest modulation of luminance, and therefore the strongest visual input, was indicated to be orthogonal to the Gabor patch orientation (18).

## Methods

### Participants

Twelve participants, four males and eight females, with a mean age of 25.3±1.5 took part in the study. All participants were healthy, had normal or corrected to normal vision and were naive to the purpose of the study. The study protocol followed the Declaration of Helsinki. In addition, the study was approved by the ethics committee of the Faculty of Medicine of the University Tuebingen and the signed informed consent was obtained from all participants prior to the experiment. All participants were recruited from University Tuebingen.

### Stimuli and procedure

In this study all participants were required to sit with their head rested on a chin rest and forehead bar during the experiment, while no response to the given stimulus was requested. Additionally, the head-fixation setup was equipped with a sponge on both sides of the head, to defuse any undesired head movements. Room lights were turned off, while the luminance of the LCD monitor (VIEWPixx, VPixx Technologies Inc., Saint Bruno, Quebec, Canada;) was set to default luminance L=20cd/m^2^. The monitor was featured by a pixel resolution of 1920 x 1200, temporal refresh rate of 120Hz and was placed in a distance of 70cm form a participant. Prior to every measurement a nine-point calibration and its validation was performed, resulting in comparable quality of every measurement. For microsaccades stimulation, a spatially oriented pattern with sinusoidal change in luminance - Gabor patch (18) and a fixed contrast level of C=0.5 according to the Equation (1), was used. For testing the potential impact of spatial frequency and the spatial orientation of the Gabor patch, four different frequencies of 0.5, 4.0, 11.0 or 22.0 cycles/degree (cpd) and two orientations of 45° and 135° were included. The orientation of Gabor patch was randomized within measurement. The visual stimulus was circular in shape, of a size of 3°.

**Figure 1.**
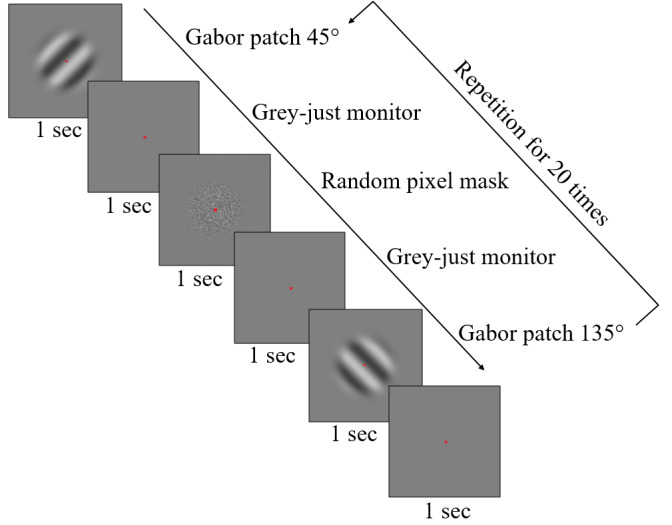
Workflow of the experiment. All five measurements per spatial frequency of the Gabor patch consisted of 20 presentations of the grating in both, 45° and 135° in a random order. Every Gabor patch was followed by a grey blank monitor to maintain the same baseline for both patterns. In addition, a noise mask of the same mean luminance was included resulting in cancel any retinal afterimages. A red dot was present across all patterns to help the participant to maintain the fixation in the desired area.

Before every presentation of a Gabor patch the monitor was set to grey with a red fixation mark in the centre of a 15arcmin size, resulting a baseline condition without any spatial visual information. In the same fashion, the fixation mark was included in the grating stimuli as well, to assist a participant to maintain fixation in the desired area. Furthermore, for avoiding any undesired afterimages a noise mask of the same size was included in the workflow. This mask was created by pixel randomization, by changing the spatial location of every pixel of the Gabor patch and thus resulting in the same mean luminance. Additionally, all stimuli were presented through a Gaussian window, to smooth the edges in order to avoid the enhanced edge detection by the visual system (38).The entire procedure is shown in Figure (1). The stimuli and the workflow were programmed in a matrix-based software (MATLAB R2018b, MathWorks, Natick, MA, USA) and the Psychtoolbox-3 extension (39, 40).

**(1):**
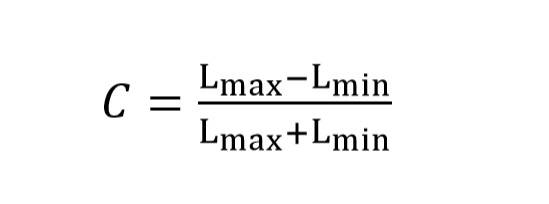


In Equation (1) Lmax and Lmax represents the maximal and minimal luminance of a visual stimulus. Every participant underwent 5 measurements, resulting in 200 observations of the Gabor patch observations per spatial frequency. Both types of the visual stimuli, as well as the grey-just monitor, were presented for the same time, t=1sec. Furthermore, this workflow was performed under both monocularly and binocularly stimulated conditions, while the examining under monocularly stimulated conditions was realized by covering the left eye with an infrared (IR) filter to forestall any visual stimulation for this eye. The transmission characteristics of the IR filter (ePlastics, San Diego, CA, USA) was T > 90% for λ > 800nm and thus resulting in eye tracking always in a binocular fashion, as the infrared light (λ =850 – 940nm) of the eye tracker (EyeLink 1000 Plus, SR Research, Ottawa, Canada) passed the filter. The sampling frequency of the eye tracker was set to 1000 Hz in both stimulation conditions.

### Analysis of the fixational eye movements

Prior to the detection of microsaccades all blinks were removed with a buffer of 50ms before and after the blink to protect the data from semi-blinks and blink-related artefacts. Blinks were detected for pupil size equal to zero. Further filtration of microsaccades was performed using the original version of Engbert’s velocity-based algorithm in the same way for both, monocularly and binocularly stimulated conditions (16). This could be done because of the tracking in a binocular fashion (see Stimuli and procedure). Applying the Engbert’s algorithm, the time series of a gaze position were firstly transformed to velocities as a moving average over 5 data samples, resulting in the noise suppression (16). Secondly, this algorithm works with the velocity thresholds obtained by application of the median estimator to the time series separately for horizontal and vertical components. Detection thresholds were computed separately for each trial and relatively to the noise level, as proposed by (16).

Next, the horizontal and vertical components were multiplied by a model’s free parameter, that was set to a usual value, (λ=6) (16). Because microsaccades are traditionally defined as a binocular occurring events (16, 24, 30, 27, 1), the Engbert’s velocity-based algorithm takes this knowledge into account by including the time overlapping criterion, that could be considered in both of the stimulation conditions (16). To prevent the analysis from overshoots, which may be examined as a separate eye movement events, a least time between two microsaccades was conservatively set to 50ms (19). Lastly, just microsaccades smaller than 1° and larger than 1arcmin in their amplitude and longer than 5ms in their duration were taken for the further analysis. For the analysis of microsaccades, MATLAB R2018b (MathWorks, Natick, USA) was used.

### Data computations and statistics

If necessary, prior to the statistical assessment the particular data were tested for their normal distribution to avoid any drawbacks coming from the statistical computation. The assessment of normality was performed by Anderson-Darling test in the MATLAB environment. All statistics was performed for the default level of significance 5%.

#### Main sequence

Firstly, the microsaccadic peak-velocity and amplitude relationship – main sequence, as shown by Bahill, Clark, & Stark, (42); Otero-Millan et al., (28); Zuber, Stark, & Cook, (41), was evaluated as a linear regression (43). This has been done separately, with respect to the two stimulation conditions. Since it has been known, that the microsaccades follow the main sequence pattern, this analysis was done to justify that the stimulation conditions did not change its typical appearance. Moreover, the statistical comparison of amplitude and peak velocity in both eyes was done as testing for potential binocular disconjugacy of microsaccades (44). This testing was performed for both stimulation conditions using a non-parametric paired t‑test (Wicoxson rank-sum test).

#### Directional distribution of microsaccades

The directions of microsaccades were computed for every tracking sample (1ms). This was done by collecting the gaze position in every sample over the time length of every microsaccade and consequently treating each sample position exclusively. As originally the units were in pixels, the necessary conversion to degrees was performed by translating all gaze positions to polar coordinates. This approach resulted in a detailed description of directional distribution for all fixational saccades, as depicted on Figure (3). For the further testing, since there have been presentations of the stimulus without any microsaccadic response, the mean microsaccadic direction was calculated. Prior to the calculation, flipping of directions was performed by adding π to all direction smaller than –π/2 and subtracting a π from directions a larger then π/2. From that flipped samples the absolute value was taken. The actual microsaccadic mean direction was calculated for each measurement for all participants using circular mean function included in circular statistics toolbox for MATLAB (45). Testing whether the mean directions vary for the Gabor patch orientation or stimulation conditions the non-parametric two-way ANOVA (Friedman’s test) was used with factors of the Gabor patch orientation and its spatial frequency. Furthermore, as the potential influence of either monocularly or binocularly stimulated conditions was tested, the non-parametric two-way ANOVA (Friedman’s test) was performed with factors of spatial frequency of the Gabor patch and the two stimulation conditions, regardless the orientation of the grating.

#### Microsaccadic rate signatures

The modulation of the microsaccadic rate over time, rate signature, was calculated as proposed by Rolfs et al., (2). First the Dirac function was applied to all times of microsaccadic events, given the rate by temporal averaging by applying a window function. To obtain the desired rate modulation curve, the decay parameter α=1/30 was employed, comparably to Gao, Yan, & Sun, (46); Rolfs et al., (2). Finally, the mean microsaccadic rate modulation over time was calculated by computing individual rate modulations and averaged across participants. The baseline of microsaccadic rate was calculated from the grey monitor containing just the centrally located red dot, that was taken as an irrelevant stimulus. The baseline was calculated for 300 ms before stimulus onset, that corresponds to time (t=0 ms). The curve of microsaccadic rate signature was created separately for each spatial frequency of the Gabor patch both stimulation conditions (see Results Figure (4) and Figure (5)). To analyse the shape of rate signature curve, the current study employed the derivative approach as shown by Henrich et al., 2004. Consequently, for the correlation of the averaged rate signature curves in the two stimulation conditions, the difference between adjacent rates has been taken across the particular averaged curve. This resulted in obtaining of the actual changes in microsaccadic rate signature curve, which were directly reporting the shape (47). These derivatives were compared for the two stimulation conditions, separately for each of spatial frequency of the grating. Additional testing of the rate signature curves was performed for comparison of the time properties and the amplitude. For this, individual rate signature curves were calculated with respect to the spatial frequency of the grating and a stimulation condition. To test the time properties of the rate signature curve given for monocularly and binocularly stimulated conditions, the time of both peaks, the minimum in microsaccadic inhibition valley and the maximum microsaccadic enhancement, after stimulus onset were found for all subjects. The search for the times of peaks and amplitudes was performed with MATLAB finding peaks function. This resulted in a two-dimensional-coordinate outcome reporting the amplitude and time of a peak. Given times for the inhibition and enhancement have been clustered according to the spatial frequency of the grating and the two stimulation conditions and tested using the two-way ANOVA. To statistically test the effect of spatial frequency of a grating and the stimulation conditions on the rate signature amplitude, the Fiedman’s test was used. To test the accuracy of the eye tracker, the validation offset of the eye tracker for the left and right eye was compared in both stimulation condition. The validation offset of the eye tracker was tested over the first measurements of every spatial frequency by the paired t‑test.

## Results

### Eye tracking quality

As proposed by Nyström et al. (48) or more recently by Ehinger et al. (49) , the eye tracking data quality is here reported first considering the accuracy and precision of the calibrated eye tracking validation and data loss. The spatial accuracy of the eye tracking was analysed using the validation offset provided by the Eye Link 1000 plus as a mean value from all nine calibration points and all participants. The precision of the eye tracker was calculated as a root-means-square error of the validation offset values from the participants throughout the different measurements. Given the data loss, the proportional time in which the signal was lost, due to a blink or other disability for pupil detection, was calculated for the time of stimulus presentation.

Monocularly stimulated conditions revealed a mean accuracy of 0.35±0.21 deg for the right eye and 0.36±0.31 deg for the left eye, which was covered by the infrared filter (p = 0.79, t-test). The precision was 0.41 deg for the right eye and 0.47 deg for the left eye. Binocularly stimulated conditions showed a mean accuracy value of 0.27±0.17 deg for the right eye and 0.31±0.24 deg for the left eye. The statistical comparison did not reveal significance (p = 0.35, t-test). The precision was 0.32 deg for the right eye and 0.39 deg for the left eye. The resulting proportional data loss was 3.0% (2.3%) for the right eye under monocular (binocular) stimulated conditions, while 3.5% (2.6%) for the left under monocular (binocular) stimulated conditions.

### Main sequence

**Figure 2.**
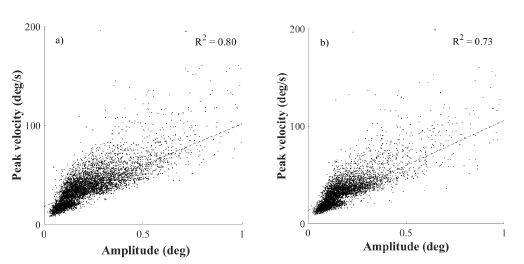
The row of figures shows the paradigm of the microsaccadic main sequence. Subplots a) and b) are plotted for the microsaccdes stimulated by the Gabor patch, whereas a) is for monocularly and b) for binocularly stimulated conditions. Every upper right corner of a particular plot shows the goodness of fit of the linear regression.

The results shown in Figure (2(a-b)) affirm that the distinct stimulation conditions did not change the typical appearance of a main sequence paradigm (28, 41, 42). The trend of linear regression in main sequence was shown for both, monocularly and binocularly stimulated conditions for microsaccades triggered by the spatially oriented grating. Linear regression revealed R^2^=0.80 for the monocularly stimulated conditions, and R^2^=0.73 for the binocularly stimulated conditions covering the data obtained from all spatial frequencies of the Gabor patch.

In the current study, the mean microsaccadic peak velocity of the right eye was 38.1±22.9 deg/s (33.8±18.6 deg/s) in monocularly (binocularly) stimulation. In the left eye the mean microsaccadic peak velocity was found as 38.7±19.9 deg/s (33.6±17.8 deg/s) in the same order. Additionally, the mean microsaccadic amplitude of the right eye was 0.21±0.14 deg (0.18±0.13 deg) in monocualrly (binocularly) stimulation condition.

For the left eye the mean amplitude of microsaccades was 0.22±0.14 deg and 0.19±0.13 deg in the same order. The statistical testing revealed no significant differences for both eyes in both, amplitude (p=0.12, rank‑sum test) and peak velocity of microsaccades (p=0.06, rank-sum test) in monocular stimulation. The same result was obtained for binocular stimulation for which the testing statistics revealed (p=0.55, rank-sum test) for the microsaccadic amplitude and (p=0.88, rank-sum test) for the peak velocity.

### Directional distribution of microsaccades

**Figure 3.**
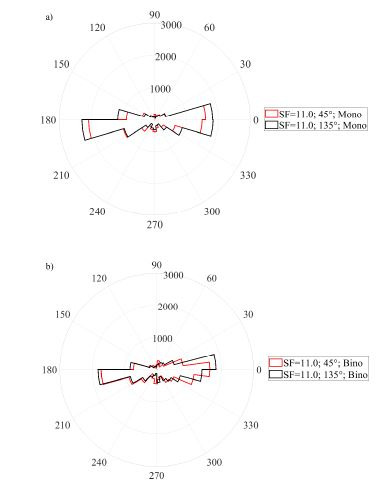
The two figures show the distribution of the microsaccadic orientations, which were taken over all samples in stimulation of a Gabor patch featured by SF=11cpd in respect to its orientation. Subfigure (a) shows the distribution for monocularly stimulated conditions, whereas subfigure (b) shows the distribution for binocularly stimulated conditions.

According the assumption that the directions of microsaccades will be influenced by the orientation of a Gabor patch, since different visual input has been found in different directions (18), the analysis was testing the microsaccadic directional distribution in respect of the orientation and spatial frequency of the stimulus, as shown on Figure (3). Furthermore, the comparative analysis was done for the two stimulation conditions, since a potential influence of presence of binocular vision was expected, according to the previous research. Prior to the statistical analysis the microsaccadic efficient direction was calculated, using the circular statistics (45) (see Data computations and statistics) and thus the mean direction of a signal was found. All these directions and their standard deviations are shown in the Table1 and Table2. These calculations were performed considering visual stimulus features (orientation and spatial frequency) as well as the stimulation conditions, exclusively. Further statistical testing revealed that the spatial orientation of the Gabor patch appeared to have no significant impact on the direction of microsaccades in the monocularly stimulated conditions as the Friedman’s test revealed (χ^2^(3)=2.65; p=0.45) as a well as in the binocularly stimulated conditions (χ^2^(3)=1.16; p=0.76) over all spatial frequencies. In testing of the potential difference in microsaccadic mean direction for the stimulation conditions, regardless the orientation of the grating, no significant influence of the stimulation conditions was found, as the Friedman’s test revealed (χ^2^(3)=0.71; p=0.81). To sum up, considering the mean microsaccadic direction, the results showed that microsaccades remained preferably horizontally oriented. This finding was shown to be consistent for all used spatial irrespective to the spatial orientation of a grating. In order to test the effect of the stimulation condition, no significant changes in the microsaccadic mean direction was observed.

**Table 1.** Mean directions of microsaccades plotted for all used spatial frequencies and the orientations of Gabor patch (GP) exclusively considering monocularly stimulated conditions.

SF(cpd)	0.5		4.0		11.0		22.0	
GP orient. (°)	45	135	45	135	45	135	45	135
mean(°)	22	20	22	22	24	21	23	22
SD (°)	22	21	23	23	24	22	24	23

**Table 2.** Mean directions of microsaccades plotted for all used spatial frequencies and the orientations of Gabor patch (GP) exclusively considering binocularly stimulated conditions.

SF(cpd)	0.5		4.0		11.0		22.0	
GP orient. (°)	45	135	45	135	45	135	45	135
mean(°)	23	26	24	26	27	24	26	27
SD (°)	22	25	25	25	25	23	25	25

### Microsaccadic rate signatures

**Figure 4.**
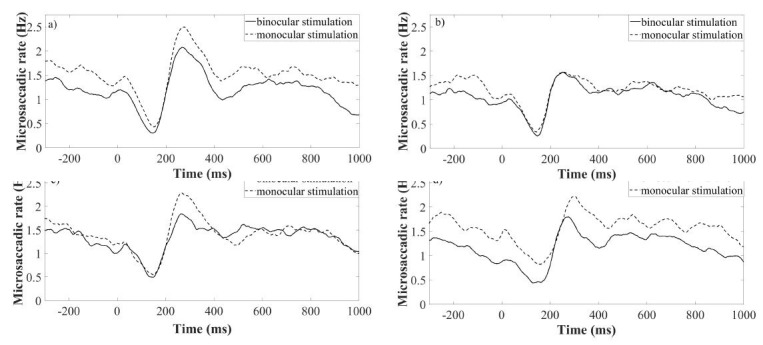
Rate signatures triggered by various density of a grating under monocularly (dotted lines) and binocularly (continuous lines) stimulated conditions. Each of the subfigures represent one spatial frequency of a grating. Subfigure a) is plotted for sf=0.5 cpd, b) 4.0 cpd c) 11.0 cpd, d) 22.0 cpd. Time = 0 corresponds to the stimulus onset.

**Figure 5.**
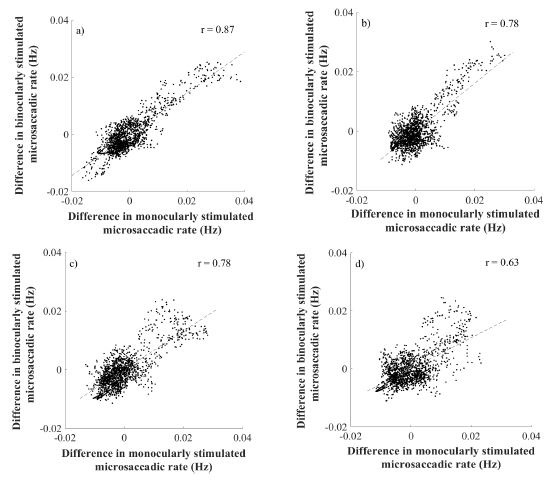
The figure a) represents the correlation for SF=0.5cpd, b) represents the correlation for SF=4.0cpd, c) represents the correlation for SF=11.0cpd and figure d) represents the correlation for SF=22.0cpd. Every upper right corner shows the index of correlation.

The data analysis showed the rate signature curve for all used spatial frequencies of the Gabor patch pattern. Furthermore, this finding was obtained in both, monocularly and binocularly stimulated conditions, as shown in Figure (4(a-d)). To analyse the identity of microsaccadic rate signature curves triggered under monocularly and binocularly stimulated conditions (see Figure (4a-d)), first the difference between adjacent rates across the averaged microsaccadic rate signature curve was taken for all used spatial frequencies separately. The consequent Pearson correlation disclosed a linear correlation in microssaccadic rate signature changes of both stimulation conditions over a wide range of spatial frequencies of the grating (r_all_>0.62; p_all_<0.0001) as depicted on the Figure (5). Furthermore, the time of microsaccadic inhibition and enhancement was compared for the given density of the grating of the two stimulation conditions across all participants. The two‑way ANOVA showed no significant time shift in, microsaccadic inhibition after stimulus onset as the effect of spatial frequency was found (F(3,88)=1.46; p=0.23) and the effect of stimulation condition (F(1,88)=1.91; p=0.17), the interaction of these parameters was found as (F(3,88)=0.42; p=0.74). For microsaccadic enhancement the two-way ANOVA showed no significant time shift as well, as the effect of spatial frequency was found (F(3,88)=1.98; p=0.12) and the effect of stimulation condition (F(1,88)=0.99; p=0.32), the interaction of these parameters was found as (F(3,88)=0.29; p=0.83). Thus the matching timing of those rate signature parameters is expected in both stimulation conditions. In testing the effect of stimulation conditions and spatial frequency of a Gabor patch on the rate signature amplitudes, Friedman’s test revealed no significant change over all spatial frequencies in the two used stimulation conditions for both, amplitude of microsaccadic inhibition (χ^2^(3)=1.0; p=0.80) and microsaccadic enhancement (χ^2^(3)=1.62; p=0.66).

## Discussion

In the current study, the analysis of the directional distribution and the rate signatures of microsaccades was performed under monocularly and binocularly stimulated conditions, while under monocular stimulation the left eye was covered by an IR filter. The evaluation of the microsaccadic metrics was done in respect of spatial characteristics of a spatially oriented grating - Gabor patch. As these metrics, the orientation and spatial frequency were taken into consideration. In both stimulation conditions, microsaccades followed the typical pattern for peak velocity and amplitude relationship – main sequence (28, 41, 50). Additionally, the current study compared the microsaccadic amplitude and peak velocity in both eyes in both stimulation conditions. The slight inequality in the microsaccadic amplitude and peak velocity of the left and right eye in both stimulation conditions may come from a potential binocular disconjugacy of microsaccades as found by Shaikh & Ghasia, (44) . Nonetheless, such a difference was not considered as a reason for claiming microsaccades as not conjugate and thus the protocol of the current study followed the approach to see microsaccades as a conjugate eye movement events respecting the Hering’s law of equal innervation (31).

### Directions of microsaccades

The hypothesis of the current study covered an assumption that microsaccades will follow the orthogonal direction to the orientation of the centrally located spatially oriented pattern preferably. As it was already shown, the orthogonal direction to the orientation of such a grating is characterized by the highest modulation of luminance, which could be described by a sine function, and therefore is this direction expected to provide the maximum of visual input (18). Nonetheless, in the current study, microsaccades were found as mainly horizontally oriented with a small vertical component. As it was found by Engbert & Kliegl, (16); Hermens & Walker, (23); Meyberg, Werkle-Bergner, Sommer, & Dimigen, (51) microsaccades occur as preferably horizontally oriented and towards a peripheral cue. This finding was explained as a relationship between the location of an attentional cue and microsaccadic orientation. As the visual stimuli appeared always in the center of a screen, the attention of all participants was expected to be at that area as well (12). Furthermore, for centrally located visual stimuli is the outcome of mainly horizontally oriented microsaccades in accordance with Kloke et al., (30) . As it was already speculated, mainly horizontally oriented microsaccades are assumed to occur for their potential purpose of binocular correction of the disparity (12). Moreover, the current study found matching directional distribution by taking into comparison the mean microsaccadic directions for monocularly and binocularly stimulated conditions. On the one hand, this finding is again assumed to be explained by the attentional location, that was always in the centre of the screen in both stimulation conditions (16, 21, 23). On the other hand, as shown by (23, 30) the microsaccadic directional distribution may vary with the implementation of the term of monocular microsaccades into the analysis, as the larger number of vertically oriented microsaccades was found considering fixational saccades as monocular events. As the current study protocol took microsaccades strictly as a binocular phenomenon with a respective microsaccadic time overlapping on both eyes in both stimulation conditions, coming from the original version of Engbert’s algorithm (16) the finding of preferably horizontally oriented microsaccades is then in correlation with both mentioned studies of Hermens & Walker, (23) and Kloke et al., (30) when considering matching microsaccadic events on both eyes. Furthermore, the outcome of preferably horizontally microsaccades could be explained by assumption that those fixational saccades share common oculomotor generator (41, 52, 53). Recently it was shown, that people perform normal saccades as mainly horizontal orientation when observing natural images (54). Additionally, it has been shown by Foulsham, Kingstone, & Underwood, (55) that willing saccades to be oblique, following a tilt of an image and thus to be mainly horizontally oriented relatively to the image orientation. In addition Wismeijer & Gegenfurtner, (56) showed saccades to mainly follow the direction of a spatially oriented grating. However, since micosaccades have been understood as involuntary fixational eye movements it is expected, that their preferably horizontal orientation comes from their involuntary character, as controlling of involuntary oblique movements has been shown as questionable (12).

### Microsaccadic rate signatures

The current study found a significantly correlated behaviour of the well-known microsaccadic rate signature curve for both monocularly and binocularly stimulated conditions by taking into comparison the rate differences over the entire rate signature curve, that reported the actual change of the rate in time. This finding was expected, as it was assumed that the microsaccades share common neural mechanism with normal saccades for their creation (41, 52, 53). Hence, microsaccades can be understood as conjugate eye movements, following the Hering’s law of equal innervation (31), resulting in synchronized microsaccadic events in both eyes in both stimulation conditions. This assumption was affirmed by comparable amplitudes of microsaccades in both eyes in the current study.

Recently, the microsaccadic rate signatures have been found to be sensitive to small changes in contrast of visual stimuli ranging from 1.3% to 4%. In detail, the change in contrast for a Gabor patch of spatial frequency of 0.33cpd caused a distinct change in the amplitude of the microsaccadic rate signature curve (19). Additionally, the similar outcomes were shown by Bonneh et al., 2015, where the microsaccadic rate signature curve revealed decreasing amplitude with decreasing contrast level of a Gabor patch featured by 3.0cpd with varying contrast level from 0.8% to 25%. On top of that, the past research disclosed that the rate signature varies over different contrast levels for a visual stimuli of circular shape, not defined by any preferred direction of luminance modulation (2). In the current study, distinctly to Scholes et al., (19), one single contrast level of the visual stimuli was taken; C=0.5, however the spatially oriented pattern was featured by a wider range of spatial frequencies (0.5; 4.0; 11.0; 22.0) cpd, compared to Bonneh et al., (20). In respect to the previous research the microsaccadic inhibitions shown by Scholes et al., 2015 for spatially oriented patterns are about 30ms delayed in comparison to those ones obtained for the Gabor patch in the current study. It is assumed that this could be explained by much higher contrast level in the current study, thus the visual input is expected to be more vivid. This assumption is confirmed by Bonneh et al., (20), as the comparable time of microsaccadic inhibition was found for the visual stimuli of 25% in the level of contrast. Additionally, to this finding, it was already proposed, that microsaccadic rate modulation is highly dependent on the visibility of presented visual stimulus (57, 58). Hence, the connection to the distinction in rate signature curves over different contrast levels of a visual stimulus.

Furthermore, in accordance to the previous research, no significant differences in timing and amplitude of microsaccadic rate signature inhibition and enhancement among the used spatial frequencies of the Gabor patch was found. This finding is notably comparable with Bonneh et al., (20), for smaller range of spatial frequencies of a spatially oriented patterns, however.

In connection to the previous research, that proposed to use the shape of a microsaccadic rate signature for an objective estimation of visual performance, like contrast sensitivity (20, 19), the current study extended the applicability of microsaccadic rate signature into the clinical practise. The current study showed the evidence of correlated rate signature curves under distinct stimulation conditions in healthy subjects and thus proposes to use monocularly stimulated microsaccadic rate signature as a tool for estimation of visual sensitivity following the clinical attitude, since such a metric of visual performance as contrast sensitivity has been measured under monocular conditions in the clinical environment.

## Limitations

To keep the comparable quality of every measurement a nine-point calibration was performed. Despite this fact, it should be still considered that microsaccades are tiny in the amplitude, thus even a usual inaccuracy in the calibration may result in a potential error. This may result in eye tracking artefacts, wrongly labelled as eye movements. Another considerable limitation is setting a threshold of amplitude of microsaccades, setting a minimum of microsaccadic length, or decay parameter for the rate signature analysis (α), or the parameter for the velocity based algorithm (λ) (16) as it is a non-objective method. These parameters have been chosen in regards to the previous research, however they may vary across researches and therefore the outcomes may not be exactly comparable. According to this problem, another approach like machine learning software for detecting microsaccades may be considered, in which case this disadvantage is solved (1). At the last point all measurements were conducted under a head-fixed position, by adding sponges on both sides of the head-rest. This condition is far from the natural viewing the scenario, and therefore the potential influence on the revealed data is expected.

## Conclusion

In conclusion, the current study has found the direction of microsaccades as preferably horizontally oriented independently to the orientation of the Gabor patch, as well as for the spatial frequency of that grating. Furthermore, the results presented in this study suggest, that the mean direction of microsaccaedes does not change under either monocularly or binocularly stimulated conditions. Therefore, this study could not report a finding of microsaccades to be sensitive to distinct spatial orientation of a grating or distinct stimulation condition and thus could not fulfil the hypothesis to possibly employ the microsaccadic directions in future contrast sensitivity testing. However, for the curves of microsaccadic rate signature a significant correlation was found for monocularly and binocularly stimulated microsaccades across a wide range of spatial frequencies of a Gabor patch. In connection to the previous studies, proposing to use the microsaccadic rate signature curve as a useful metric for estimating visual performance, as for instance contrast sensitivity by varying the contrast of a grating stimuli, the current study shows a methodological correction to the previous research resulting in a possible usage of monocularly stimulated microsaccadic rate signatures. These have been shown to behave in a similar way in healthy subjects, in two distinct stimulation conditions, while in both following the fundamental understanding of microsaccades as a binocular phenomenon.

To conclude, this study proposes to analyse microsaccades under monocularly stimulated conditions, since clinical metrics of visual performance have been usually estimated under monocular viewing conditions as well. In such a way the estimation of visual performance from microsaccades could follow the clinical standard.

### Ethics and Conflict of Interest

The authors declare that the contents of the article are in agreement with the ethics described in http://bib-lio.unibe.ch/portale/elibrary/BOP/jemr/ethics.html.

This work was done in an industry-on-campus-cooperation between the University of Tuebingen and Carl Zeiss Vision International GmbH. Author P.E. declares no potential conflict of interest. A.L., K.R. and S.W. are employed by Carl Zeiss Vision International GmbH and are scientists at the University Tuebingen.

### Acknowledgements

Funding was received from Eberhard-Karls-University Tuebingen (ZUK 63) as part of the German Excellence initiative from the Federal Ministry of Education and Research (BMBF). Further funding received from Deutsche Forschungsgemeinschaft and Open Access Publishing Fund of University of Tuebingen.
